# Vascular, Cognitive, and Psychomental Survey on Elderly Recycling Volunteers in Northern Taiwan

**DOI:** 10.3389/fneur.2018.01176

**Published:** 2019-01-10

**Authors:** Guei-Chiuan Chen, Pei-Ya Chen, Yu-Chin Su, Cheng-Lun Hsiao, Fu-Yi Yang, Po-Jen Hsu, Shinn-Kuang Lin

**Affiliations:** ^1^Stroke Center and Department of Neurology, Taipei Tzu Chi Hospital, Buddhist Tzu Chi Medical Foundation, New Taipei City, Taiwan; ^2^School of Medicine, Tzu Chi University, Hualien, Taiwan

**Keywords:** carotid atherosclerosis, mild cognitive impairment, mood disorder, recycling work, vegetarian, volunteer

## Abstract

**Background:** Stroke and dementia represent frequent causes of psychophysical and socioeconomic burdens. We conducted a vascular, cognitive, and psychomental survey involving elderly volunteers at community-based recycling stations in Northern Taiwan.

**Methods:** Recycling volunteers aged ≥60 years were surveyed. We recorded seven parameters, namely (1) body mass index (BMI), (2) fasting glucose, (3) fasting cholesterol, (4) ankle-brachial index (ABI), (5) carotid duplex sonography, (6) five-item Brief Symptom Rating Scale (BSRS-5) score, and (7) eight-item Interview to Differentiate Aging and Dementia (AD8). During the carotid duplex study, we measured the carotid intima-media thickness (CIMT) and the carotid total plaque score (CTPS) of the common and internal carotid arteries.

**Results:** In total, 985 subjects (mean age: 70.8 years) participated in this study. Among these, 81% were women, and 52% were vegetarians. The average ABI, CIMT, and CTPS were higher in men, whereas women had higher cholesterol levels and BSRS-5 scores. Obesity, hypertension, hyperglycemia, and hyperlipidemia were present in 21, 38, 9, and 27% of all subjects, respectively. Carotid plaques with mild (CTPS 1–5), moderate (CTPS 5.1–10), and severe (CTPS > 10) atherosclerosis were detected in 45, 16, and 7% of the subjects, respectively. Mild cognitive impairment (AD8 > 2) was observed in 13% of the subjects, whereas moderate mood disorder (BSRS-5≧10) was observed in only 1% of subjects. Vegetarians had a lower BMI, systolic blood pressure (SBP), cholesterol, CIMT, and CTPS than did non-vegetarians. Substantial predictors of severe atherosclerosis were advanced age (>70 years), male sex, history of heart disease, hyperlipidemia, and currently elevated SBP and cholesterol levels. Predictors of mild cognitive impairment were illiteracy, history of hypertension, hyperlipidemia, and moderate mood disorder.

**Conclusions:** Subclinical carotid atherosclerosis was common in elderly recycling volunteers, with 23% having moderate to severe stenosis. Vegetarians had a reduced risk of atherosclerosis. The low incidence of moderate mood disorder might indicate that recycling work enhances psychomental health. In addition, a healthier lifestyle, better mood condition, and vegetarian diet might contribute to lower incidence of mild cognitive impairment.

## Introduction

Stroke and dementia are the most common neurological causes of psychophysical and socioeconomic burdens on the public health among elderly people. With improvements in acute stroke treatment through intravenous thrombolytic therapy (1) and adherence to the stroke guidelines established by the American Stroke Association (2) and Taiwan Stroke Society (3, 4), stroke has declined from the second to the fourth leading cause of death in Taiwan (5) over the past two decades. However, stroke remains the leading cause of disability among elderly people. Taiwan's population is aging because of a declining birth rate and an increasing life expectancy. This caused the percentage of elderly people (>65 years) to increase to 12.5% in 2015. The annual incidence of dementia has also increased, from 1.2% in people aged 65–69 years to 30.9% in those aged 90 years or older in Taiwan (6). A nationwide survey by Sun et al. revealed that the age-adjusted prevalence of all-cause dementia was 8.04% in people aged 65 years or older in 2014 (7).

Common risk factors for stroke include advancing age, hypertension, diabetes mellitus, hyperlipidemia, heart disease, obesity, immobility, cigarette smoking, and alcohol consumption (8). Other factors including depression and marital status have also been noted (9). Alzheimer's dementia and vascular dementia are the two main types of dementia. In addition to genetic factor and female gender, advancing age and low education are two confirmed risk factors for dementia in Taiwan (6). Other risk factors for dementia include lifestyle choices, depression, and medical conditions that are similar to vascular factors (10). Nuyen et al. reported that subjects who had previously experienced depression had higher severity of acute stroke and poorer prognoses after stroke (11). Chan et al. demonstrated that high cerebrovascular risk factor scores were related to an increased risk of suicide in older adults (12). A systematic review by Pompili et al. found that depression, previous mood disorder, prior history of stroke, and cognitive impairment were the primary risk factors for suicide (13). Nevertheless, most modifiable risk factors for stroke and dementia can be controlled and treated through early detection and recognition. Furthermore, treatment of stroke risk factors could reduce the risk of depression or stroke (9).

Community-based recycling work represents a special cultural phenomenon dedicated to a cleaner environment and was established by a Buddhist compassion foundation in Taiwan in 1990. Currently, more than 110,000 volunteers participate in recycling work at over 8,600 environmental stations in Taiwan's cities. In total, 46% of recycling volunteers are elderly people (>65 years) from the local community. Most people are responsible for sorting, classifying, and deconstructing recyclable trash at environmental stations, performing various related activities. Because of the Taiwanese population's religious beliefs, the number of vegetarians is relatively high, and the cigarette or alcohol consumption is relatively low among volunteers. Lifestyle choices may affect the risk of stroke, depression, and dementia. Regular physical activity from recycling work and vegetarian diet are linked to a special manner of lifestyle exhibited by recycling volunteers. To explore the prevalence rates of vascular, cognitive, and psychomental disorders among elderly volunteers, we conducted a community-based survey in northern Taiwan for the early detection of stroke and dementia risk.

## Materials and Methods

### Design and Participants

This was a prospective study conducted from May 2015 to December 2016. A health survey team organized by the stroke center of the index hospital and comprising physicians, nurses, technicians, and administrative staffs visited various districts in Northern Taiwan to conduct health examinations, of which vascular, cognitive, and psychomental surveys were the primary components. Most of the physicians and technicians in the health survey team were staff members of the stroke center; all other team members were unpaid assistants from various departments of the hospital. Volunteers participating in recycling work at community environmental stations who were aged 60 years or older were candidates for the health survey. A questionnaire on the personal education, living status, and medical history was completed by each subject. Ischemic, valvular, and dysrhythmic heart conditions diagnosed by cardiologists were reported as each patient's history of heart disease. Subjects who had followed a vegetarian diet (i.e., consuming no animal products, with or without eggs) for 1 year or longer were considered vegetarians (14–16). This study was conducted in accordance with the recommendations and was approved by the Institutional Review Board of the Taipei Tzu Chi Hospital, Buddhist Tzu Chi Medical foundation (No. 04-X11-023). All subjects provided written informed consent in accordance with the Declaration of Helsinki.

### Instruments

We recorded the following parameters: (1) body mass index (BMI; body weight divided by body height squared); (2) fasting glucose; and (3) fasting cholesterol using a one-touch CardioChek PA Analyzer (PTS Diagnostics, Indianapolis, IN, USA) with finger blood; (4) ankle-brachial index (ABI; ratio of blood pressure at the ankle to that in the upper arm) using an Omron Colin VP-1000 Plus (Omron Healthcare, Muko, Kyoto, Japan); (5) carotid duplex sonography using a portable GE LOGIQ-e (GE Healthcare, Solingen, Germany) containing a 3.3–10-MHz transducer combining real time color B-mode and pulsed Doppler imaging; (6) a 5-item Brief Symptom Rating Scale (BSRS-5) score, a self-reported measurement of psychological distress severity, scored 0–20 (higher scores indicate less favorable mental health) (17); and (7) an 8-item Interview to Differentiate Aging and Dementia (AD8), a simple test of memory, orientation, judgment, and function scored from 0 to 8 (a high score indicates less favorable cognition) (18). The BSRS-5 contains five psychopathology items: (1) feeling tense or keyed up (anxiety), (2) feeling blue (depression), (3) feeling easily annoyed or irritated (hostility), (4) feeling inferior to others (inferiority), and (5) having trouble falling asleep (insomnia). An additional question about suicidal ideation was added at the end of the questionnaire (17, 19, 20).

**Figure 1:**
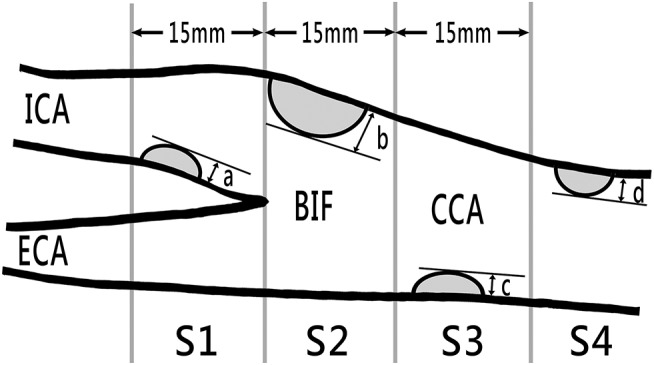
Carotid plaque scores obtained through B-mode sonography. The total plaque score was computed by summing the maximum plaque thickness (in millimeters) a in segments S1 (internal carotid artery within 15 mm distal of bifurcation), b in S2 (region of internal and distal common carotid artery within 15 mm proximal of bifurcation), c in S3 (common carotid artery 15–30 mm proximal of bifurcation), and d in S4 (common carotid artery >30 mm distal of bifurcation) on both sides. BIF, bifurcation; CCA, common carotid artery; ECA, external carotid artery; ICA, internal carotid artery.

The carotid duplex sonography was performed by trained, experienced technicians. We measured the carotid intima-media thickness (CIMT) of the distal common carotid artery on both sides. IMT was measured automatically by the ultrasound instrument as the distance between the lumen-intima and media-adventitia interfaces. A carotid plaque was defined as local thickening of the CIMT of >50% compared with the surrounding vessel wall, or a CIMT of >1.5 mm (21, 22). We also measured the carotid total plaque score (CTPS) and flow velocities for the common and internal carotid arteries. CTPS was calculated by summing the maximum plaque thickness measured on the near and far walls at each of the four divisions on both sides of the carotid arteries (Figure 1) (23).

### Definitions

A BMI of ≥27 was considered obesity. Hypertension was defined as a systolic blood pressure (SBP) of >140 mmHg on both arms. Hyperglycemia and hypercholesterolemia were defined as a fasting glucose of >126 mg/dL and a fasting cholesterol of >200 mg/dL, respectively. An ABI of < 0.9 was considered peripheral arterial disease. The CTPS results were classified as mild (CTPS 1.1–5), moderate (CTPS 5.1–10), and severe (CTPS > 10) degrees of atherosclerosis (24). A Doppler flow peak systolic velocity of >200 cm/s with plaque formation at the internal carotid artery was considered high-grade stenosis. A BSRS-5 score between 6 and 9 indicated a mild mood disorder, and a score of ≥10 indicated a moderate mood disorder (25). An AD8 score of >2 suggested mild cognitive impairment.

### Statistical Analyses

We used the free statistical software R version 3.4.3 with package “pwr” to implement power analysis in this study (26). Chi-squared and Fisher's exact tests were performed for categorical data comparisons. Differences in the means of continuous variables were tested using two-sample *t*-test and analysis of variance. Linear regression was performed to evaluate the potential effect of age on the measured variables. Factors influencing severe carotid atherosclerosis, moderate mood disorder, and mild cognitive impairment were defined using multivariate logistic regression analysis. Predictive performance levels of the variables for severe carotid atherosclerosis, moderate mood disorder, and mild cognitive impairment were compared using *C*-statistics. *P* < 0.05 was considered to indicate statistical significance. All the statistical analyses were performed using IBM SPSS Statistics for Windows, Version 24 (Armonk, IBM Corp., NY, USA).

## Results

### Subject Characteristics

A total of 985 recycling volunteers comprising 795 (81%) women and 190 men, with a mean age of 70.8 years, were examined through 29 community visits (Table 1). Approximately half (46%) of the subjects had primary school as their highest education level, whereas 23% had not received any formal education (illiteracy). More men than women had received a post-high school education. Forty-two percent of the subjects lived with a spouse and children, whereas 9% lived alone. In total, 38% of the women were widows, but only 6% of the men were widowers. Vegetarians constituted 52% of the subjects. A past history of tobacco and alcohol consumption was reported by 7 and 8% of the subjects, respectively, and was frequent among the men (32 and 18%, respectively). However, only 5% of the men currently smoked. Half (50%) of the subjects performed recycling work on three or more days per week. A history of hypertension, diabetes, heart disease, or hyperlipidemia was present in 41, 14, 18, and 21% of the subjects, respectively, with women more likely to have a history of hyperlipidemia.

**Table 1:** Demographic data of 985 volunteers.

**Characteristics**	**Total (*n* = 985)**	**Women (*n* = 795)**	**Men (*n* = 190)**	***P-*value**
Mean age (years)^*^	70.8 ± 6.8	71.0 ± 6.8	70.2 ± 6.8	0.172
Educational status				<0.001
None	227 (23%)	212 (27%)	15 (8%)	
Primary school	455 (46%)	386 (48%)	69 (36%)	
Junior middle school	134 (14%)	95 (12%)	39 (21%)	
High School	124 (13%)	78 (10%)	46 (24%)	
College/university	45 (4%)	24 (3%)	21 (11%)	
Marriage status				<0.001
Married	647 (66%)	471 (59%)	176 (93%)	
Widowed	312 (32%)	301 (38%)	11 (6%)	
Divorced	14 (1%)	12 (2%)	2 (1%)	
Unmarried	12 (1%)	11 (1%)	1 (0%)	
Accompanied living relatives				<0.001
Spouse and children	417 (42%)	301 (38%)	116 (61%)	
Spouse only	161 (16%)	104 (13%)	57 (30%)	
Children	310 (31%)	299 (38%)	11 (6%)	
Alone	87 (9%)	82 (10%)	5 (3%)	
Others	10 (1%)	9 (1%)	1 (0%)	
Vegetarian	512 (52%)	418 (53%)	94 (49%)	0.467
History of smoking/current smoker	73 (7%)/17 (2%)	13 (2%)/8 (1%)	60 (32%)/9 (5%)	<0.001
Alcohol	77 (8%)	43 (5%)	34 (18%)	<0.001
Activity of recycling work				0.171
Sporadic	217 (22%)	175 (22%)	42 (22%)	
1–2 days/week	238 (24%)	195 (25%)	43 (23%)	
3–4 days/week	154 (16%)	114 (14%)	40 (21%)	
5–7 days/week	336 (34%)	280 (35%)	56 (29%)	
Others	40 (4%)	31 (4%)	9 (5%)	
History of health condition				
Hypertension	397 (40%)	326 (41%)	71 (37%)	0.367
Diabetes	127 (14%)	104 (13%)	23 (12%)	0.809
Heart disease	179 (18%)	147 (18%)	32 (17%)	0.676
Hyperlipidemia	196 (21%)	169 (21%)	27 (14%)	0.033

Chi-square test;

* *two-sample t test*.

### Measurement Results

Table 2 shows the data obtained from 985 subjects. The average BMI was 24.4 ± 3.6 kg/m^2^. The average SBP was higher on the left arm (158 ± 26 mmHg) than on the right arm (138 ± 20 mmHg) (*P* < 0.05). No difference between the right and left sides was observed for ABI, CIMT, or CTPS. Men had a higher average ABI (1.15 ± 0.12 vs. 1.13 ± 0.10), CIMT (0.71 ± 0.18 vs. 0.67 ± 0.19 mm), and CTPS (3.75 ± 3.95 vs. 3.08 ± 3.84), whereas women had higher cholesterol levels (191 ± 36 vs. 171 ± 31 mg/dL) and BSRS-5 scores (2.5 ± 2.5 vs. 2.1 ± 2.5). Subjects on a vegetarian diet were younger and had lower BMI, SBP, cholesterol levels, CIMT, and CTPS and higher ABI than the other subjects. We performed a power analysis of cholesterol (mg/dL) for gender and vegetarian diet that shown in Table 2. We found that the power was 0.999 for cholesterol in the gender group and was 0.976 in the vegetarian diet group. The setting for the power analysis was achieved in our study for means comparison using a two-sample *t* test.

**Table 2:** Results of measured variables during health examination in 985 volunteers.

**Characteristics**	**Total (*n* = 985)**	**Gender**			**Vegetarian diet**		
		**Women (*n* = 795)**	**Men (*n* = 190)**	***P* value**	***Y* (*n* = 512)**	***N* (*n* = 473)**	***P* value**
Age (years)	70.8 ± 6.8	71.0 ± 6.8	70.2 ± 6.8	0.172	70.0 ± 6.9	71.3 ± 6.6	<0.001
Body mass index (kg/m^2^)	24.4 ± 3.6	24.4 ± 3.8	24.5 ± 3.2	0.856	23.9 ± 3.6	24.9 ± 3.7	<0.001
Systolic blood pressure (mmHg)	148 ± 25	147 ± 25	149 ± 24	0.270	146 ± 25	149 ± 25	0.006
Right arm	138 ± 20	138 ± 20	138 ± 19			136 ± 20	140 ± 19
Left arm^*^	158 ± 26	157 ± 26	160 ± 64		157 ± 26	159 ± 26	
Glucose (mg/dL)	100 ± 16	100 ± 16	102 ± 14	0.185	100 ± 17	101 ± 15	0.809
Cholesterol (mg/dL)	188 ± 36	191 ± 36	171 ± 31	<0.001	184 ± 35	193 ± 38	<0.001
Ankle-brachial index (Bilateral)	1.13 ± 0.10	1.13 ± 0.10	1.15 ± 0.11	<0.001	1.14 ± 0.09	1.12 ± 0.11	0.009
Carotid intima-media thickness (mm)	0.67 ± 0.19	0.67 ± 0.19	0.70 ± 0.18	0.011	0.66 ± 0.19	0.69 ± 0.19	0.004
Carotid total plaque score	3.2 ± 3.9	3.08 ± 3.84	3.75 ± 3.95	0.031	2.8 ± 3.7	3.6 ± 4.0	0.001
Five-item Brief Symptom Rating Scale	2.4 ± 2.5	2.5 ± 2.5	2.1 ± 2.5	0.034	2.4 ± 2.6	2.4 ± 2.4	0.625
AD8	1.1 ± 1.4	1.1 ± 1.3	1.1 ± 1.4	0.872	1.1 ± 1.3	1.1 ± 1.4	0.491

Two-sample t test;

* *p < 0.001, compared to right arm. AD8, the Eight-item Interview to Differentiate Aging and Dementia*.

### Abnormal Findings

Obesity, hypertension, hyperglycemia, and hyperlipidemia were found in 21, 9, 35, and 27% of all subjects, respectively (Table 3). Abnormal ABI was found in only 3% of the subjects. Carotid plaques were detected in 68% of the subjects, of whom 45, 16, and 7% had mild (CTPS 1–5), moderate (CTPS 5–10), and severe atherosclerosis (CTPS >10), respectively. The three subjects categorized as CTPS >10 had high-grade stenosis with a peak systolic velocity of >200 cm/s. Mood disorder (BSRS-5 ≥6) was observed in 106 subjects (11%). Moderate mood disorder (BSRS-5 ≥10) was observed in only 13 subjects (1%) and occurred more in those who performed recycling work only sporadically, without a regular schedule (7 subjects, *P* = 0.005) and in those who did recycling work ≤ 2 days per week (10 subjects, *P* = 0.025). Suicidal ideation was not reported by any of the subjects. Among the five psychomental domains, insomnia (36%) and anxiety (24%) constituted the most BSRS-5 scores. Mild cognitive impairment (AD8 >2) was observed in 125 (13%) of all subjects and occurred more in subjects who had not received any formal education (40/227, 18%) than in those with various levels of education (85/758, 11%) (*P* = 0.017). Hypercholesterolemia occurred more in women. Men had a higher percentage of hyperglycemia, CTPS ≤1 and >10. Vegetarians had a lower percentage of obesity, elevated SBP, cholesterol, abnormally low ABI, and carotid plaque formation.

**Table 3:** Results of vascular, cognitive, and psychomental survey in 985 volunteers.

**Characteristics**	**Total (*n* = 985)**	**Gender**			**Vegetarian diet**		
		**Women (*n* = 795)**	**Men (*n* = 190)**	***P* value**	**Y (*n* = 512)**	**N (*n* = 473)**	***P* value**
Body mass index (kg/m^2^) ≧ 27	207 (21%)	169 (21%)	38 (20%)	0.701	91 (18%)	116 (25%)	0.009
Elevated systolic blood pressure^*^	385 (39%)	317 (40%)	68 (36%)	0.298	183 (36%)	202 (43%)	0.026
Elevated glucose^†^	86 (9%)	62 (8%)	24 (13%)	0.044	53 (10%)	33 (7%)	0.071
Elevated cholesterol^‡^	279 (28%)	253 (32%)	26 (14%)	<0.001	117 (23%)	162 (34%)	<0.001
Ankle-brachial index < 0.9	29 (3%)	22 (3%)	7 (4%)	0.477	9 (2%)	20 (4%)	0.024
Carotid duplex sonography				0.013			<0.001
No plaque	316 (32%)	271 (24%)	45 (34%)	0.006	181 (35%)	135 (29%)	0.024
CTPS 1.1-5 (mild)	446 (45%)	355 (45%)	91 (48%)		241 (47%)	205 (43%)	
CTPS 5.1–10 (moderate)	159 (16%)	124 (16%)	35 (18%)		61 (12%)	98 (21%)	<0.001
CTPS >10 (severe)	64 (7%)	45 (6%)	19 (10%)	0.034	29 (7%)	35 (7%)	
Five-item Brief Symptom Rating Scale				0.353			0.252
BSRS-5 score <6 (normal)	879 (89%)	705 (89%)	174 (91%)			450 (88%)	429 (91%)
BSRS-5 score 6–9 (mild)	93 (10%)	80 (10%)	13 (7%)		53 (10%)	40 (8%)	
BSRS-5 score ≧ 10 (moderate)	13 (1%)	10 (1%)	3 (2%)		9 (2%)	4 (1%)	
AD8 >2	125 (13%)	100 (13%)	25 (13%)	0.809	66 (13%)	59 (12%)	0.849

Chi-square test;

* systolic blood pressure > 140 mmHg on both arms;

† fasting glucose > 126 mg/dL;

‡ *fasting cholesterol > 200 mg/dL*.

*CTPS, carotid total plaque score; BSRS-5, the Five-item Brief Symptom Rating Scale; AD8, the Eight-item Interview to Differentiate Aging and Dementia*.

Positive linear correlations of age with SBP, CIMT, CTPS, and AD8 and negative correlations of age with fasting cholesterol and ABI were found (Table 4). Positive linear correlation of CTPS with SBP, fasting glucose, CIMT, and AD8 and negative correlation with ABI were observed. A positive linear correlation of BSRS-5 with SBP and a negative correlation with BMI were found. Univariate analysis also revealed that mean SBP was significantly higher in volunteers with moderate mood disorder than in those without (151 mmHg vs. 138 mmHg, *P* = 0.018). A strong positive linear correlation of AD8 with BSRS-5 was observed. Additionally, positive linear correlations of BMI with SBP (*R*^2^ = 0.027, *P* < 0.001) and fasting glucose (*R*^2^ = 0.016, *P* < 0.001) were observed (not shown in Table 4).

**Table 4:** Simple linear regression analyses of age, TCPS, BSRS-5, and AD8 with measured variables in 985 volunteers.

	**Age**			**CTPS**			**BSRS-5**			**AD8**		
**Dependent variables**	**Coefficient**	***R^**2**^***	***P-*value**	**Coefficient**	***R^**2**^***	***P-*value**	**Coefficient**	***R^**2**^***	***P*-value**	**Coefficient**	***R^**2**^***	***P-*value**
Age	–	–	–	0.607**x**	0.120	<0.001	0.100**x**	0.000	0.252	0.366	0.005	0.023
Body mass index	0.001**x**	0.000	0.935	0.036**x**	0.000	0.231	−0.108**x**	0.005	0.021	−0.310**x**	0.000	0.723
Systolic blood pressure	0.749**x**	0.041	<0.001	1.080**x**	0.045	<0.001	0.517**x**	0.004	0.043	0.655**x**	0.002	0.159
Fasting glucose	−0.005**x**	0.000	0.952	0.508**x**	0.015	<0.001	−0.191**x**	0.001	0.402	−0.881**x**	0.076	0.036
Fasting cholesterol	−0.448**x**	0.007	0.026	0.378**x**	0.000	0.279	−0.147**x**	0.000	0.781	1.430**x**	0.003	0.149
Ankle-brachial index	−0.001**x**	0.003	0.018	−0.003**x**	0.011	0.001	−0.001**x**	0.000	0.693	0.001**x**	0.000	0.735
Carotid IMT	0.004**x**	0.021	<0.001	0.014**x**	0.007	<0.001	−0.000**x**	0.000	0.989	−0.001**x**	0.000	0.795
CTPS	0.198**x**	0.120	<0.001	–	–	–	0.082**x**	0.003	0.102	0.302**x**	0.011	0.001
BSRS-5	0.013**x**	0.000	0.252	0.033**x**	0.003	0.102	–	–	–	0.611**x**	0.109	<0.001
AD8	0.014**x**	0.005	0.023	0.036**x**	0.011	0.001	0.179**x**	0.109	<0.001	–	–	–

*IMT, intima-media thickness; CTPS, carotid total plaque score; BSRS-5, Five-item Brief Symptom Rating Scale; AD8, Eight-item Interview to Differentiate Aging and Dementia*.

Multivariate logistic regression analysis revealed the following significant predictors of severe carotid atherosclerosis (CTPS >10): age ≥70 years, male sex, history of heart disease, history of hyperlipidemia, and elevated SBP and cholesterol levels (Table 5). A *C*-statistic of 0.752 for detection of severe carotid atherosclerosis was estimated from a fit model of these six significant predictors obtained from the regression analysis summarized in Table 5. Significant predictors of moderate mood disorder (BSRS-5 ≧10) were mild cognitive impairment (AD8 > 2) and activity of recycling work ≤ 2 days per week (Table 6). A *C*-statistic of 0.774 for detection of moderate mood disorder was estimated from these two predictors. Interestingly, unmarried status tended to have negatively affect on moderate mood disorder but did not reach statistical significance (*P* = 0.058). However, addition of unmarried status improved the predictive value for moderate mood disorder from 0.744 to 0.825. Significant predictors of mild cognitive impairment were: illiteracy and histories of hypertension, hyperlipidemia, and moderate mood disorder (Table 7). A *C*-statistic of 0.625 for detection of mild cognitive impairment was estimated from a fit model of these four significant predictors obtained from the regression analysis summarized in Table 7.

**Table 5:** Logistic regression of factors influencing severe carotid atherosclerosis in 985 volunteers.

**Characteristics**	**Severe carotid atherosclerosis (CTPS > 10)**	
	**OR (95% CI)**	***P-*value**
Age ≧ 70 years	3.333 (1.71–6.59)	<0.001
Male gender	2.57 (1.28–5.16)	0.008
History of hypertension	1.67 (0.95–2.94)	0.074
History of diabetes mellitus	1.21 (0.59–2.51)	0.599
History of heart disease	2.06 (1.15–3.69)	0.015
History of hyperlipidemia	1.84 (1.01–3.36)	0.048
History of smoking	1.53 (0.54–4.33)	0.420
History of alcohol drinking	0.61 (0.19–1.90)	0.390
Vegetarian food	1.14 (0.65–1.98)	0.648
Currently elevated systolic blood pressure (>140 mmHg)	2.02 (1.17–3.49)	0.012
Currently elevated glucose (>126 mg/dL)	1.38 (0.59–3.22)	0.452
Currently elevated cholesterol (>200 mg/dL)	2.15 (1.24–3.81)	0.009
Low ankle-brachial index (<0.9)	1.83 (0.55–6.10)	0.326
High body mass index (≧27)	0.89 (0.48–1.69)	0.734

*CTPS, carotid total plaque score; OR, odds ratio; CI, confidence interval*.

**Table 6:** Logistic regression of factors influencing moderate mood disorder in 985 volunteers.

**Characteristics**	**Moderate mood disorder (BSRS ≧ 10)**	
	**OR (95% CI)**	***P* value**
Age ≧ 70 years	0.98 (0.29–3.28)	0.977
Male gender	1.07 (0.27–4.18)	0.924
Illiteracy	2.99 (0.84–10.69)	0.090
Unmarried	0.13 (0.02–1.08)	0.058
Recycling activity ≦ 2 days/week	4.33 (1.15–16.27)	0.030
Mild cognitive impairment (AD8 > 2)	5.18 (1.61–16.70)	0.006

*BSRS-5, Five-item Brief Symptom Rating Scale; AD8, Eight-item Interview to Differentiate Aging and Dementia; OR, odds ratio; CI, confidence interval*.

**Table 7:** Logistic regression of factors influencing mild cognitive impairment in 985 volunteers.

**Characteristics**	**Mild cognitive impairment (AD8 > 2)**	
	**OR (95% CI)**	***P-*value**
Age ≧ 70 years	0.97 (0.64–1.48)	0.883
Male gender	1.35 (0.82–2.23)	0.242
Illiteracy	1.67 (1.06–2.63)	0.028
History of hypertension	1.58 (1.05–2.38)	0.029
History of diabetes mellitus	1.57 (0.92–2.69)	0.097
History of heart disease	1.05 (0.64–1.72)	0.845
History of hyperlipidemia	1.59 (1.02–2.47)	0.040
Currently elevated systolic blood pressure (>140 mmHg)	0.92 (0.61–1.37)	0.664
Currently elevated glucose (> 126 mg/dL)	0.87 (0.43–1.78)	0.709
Currently elevated cholesterol (> 200 mg/dL)	1.46 (0.96–2.22)	0.075
Severe carotid atherosclerosis (CTPS > 10)	0.78 (0.36–1.68)	0.523
Moderate mood disorder (BSRS-5 ≧ 10)	4.07 (1.28–13.00)	0.018

*CTPS, carotid total plaque score; BSRS-5, Five-item Brief Symptom Rating Scale; AD8, Eight-item Interview to Differentiate Aging and Dementia; OR, odds ratio; CI, confidence interval*.

## Discussion

In this group of elderly recycling volunteers, which comprised vegetarians (52%) and current smokers (2%), we found that 68% of the subjects had subclinical carotid atherosclerosis. Of these, 23% had moderate to severe carotid atherosclerosis requiring continuous follow-up examinations or medical treatment. Mild cognitive impairment was observed in 13% of the subjects and occurred more frequently in those with illiteracy and history of hypertension, hyperlipidemia, and moderate mood disorder. Mood disorder was observed in 11% of the subjects. Moderate mood disorder was present in only 1% of the subjects and occurred less frequently in those who did recycling work ≥3 days per week. Vegetarians had a reduced risk of atherosclerosis in terms of lower BMI, cholesterol level, CIMT, and CTPS and a lower percentage of elevated SBP and abnormal ABI.

Compared with some population-based studies using hospital health examinations at the patients' own expense, the population in this study was socioeconomically relatively weak. Performing, recycling work without remuneration is considered meaningful, conscientious work to protect the environment from waste pollution. The subjects of this study were predominantly women, with a high percentage of vegetarians and a low percentage of current smokers. Recycling volunteers appear to tend to have a healthy lifestyle. Women had a lower educational background and were more likely to be widowed than were men; this observation correlates with the higher average life expectancy of Taiwanese women (83.4 years) compared with that of men (76.8 years) (27). All medical care has been covered by Taiwan's National Health Insurance system since 1995; thus, most people can receive appropriate medical help in case of illness. However, some subjects were unaware of their general health condition. According to their self-reported medical history, hypertension and hyperlipidemia were the most common risk factors for atherosclerosis. The present findings indicated that the study group had relatively lower percentages of patients with elevated blood pressure (39.1%), and high glucose (8.7%) and cholesterol level (28.3%); nevertheless, this was a highly vegetarian population with a mean age of 70.8 years. Table 8 summarizes recent population-based vascular and cognitive survey studies in different Asian areas. Reported percentage of each vascular risk was from 35.5 to 78.4% for hypertension; from 13.6 to 37.9% for diabetes mellitus; and from 19.1 to 73.7% for hyperlipidemia. However, compared with the present study, the studied population with the lowest prevalence of hypertension (13.6%) and hyperlipidemia (19.1%) reported by Chou et al. was much younger (mean age 52.8 years) and the definition of hyperlipidemia was much higher (cholesterol ≧ 240 mg/dL) (28). The data in the present study might be affected by several factors, such as insufficient rest, an unsuitable location for checking blood pressure, or insufficient accuracy of the results of the one-touch finger blood test. Moreover, definitions of abnormal results might not have been similar in other studies. However, for a more effective control of risk factors, including greater compliance with medication, diet control, and regular follow-up, these results require further attention. Vegetarians have been reported to have lower BMI and blood pressure, less hypertension, and a lower incidence of diabetes than non-vegetarians (16, 33, 34). In this study, vegetarians had lower cholesterol, CIMT, CTPS, SBP, and BMI and higher ABI. No difference was found between vegetarians and non-vegetarians with respect to history of diabetes or blood glucose levels.

**Table 8:** Summary of population-based vascular and cognitive survey studies in different Asian areas.

**Characteristics**	**Present study 2018**	**Chou et al. (28)**	**Sun et al. (7)**	**Wang et al. (29)**	**Yan et al. (30)**	**Shaik et al. (31)**	**Moon et al. (32)**
Area	Taiwan	Taiwan	Taiwan	China	China	Singapore	Korea
Population	Recycling volunteers	Rural population	General population	Kailuan community	Rural population	General population	General population
No. of patients	985	1,539	10,432	3,048	1,375	832	348
Main interests	CTPS, CIMT, ABI, cognition	CTPS	Cognition	ABI, cognition	Carotid stenosis, cognition	ABI, cognition	CIMT, ABI, cognition
Inclusion age (years)	≧60	≧40	≧65	≧40	≧60	≧60	≧65
Mean age (years)	70.8	52.8	76.2	57.9	68.8	70.1	71.7
Education ≧6 years (%)	69.2	–	77.3	22.1	–	–	–
Mean BMI (kg/m^2^)	24.4	24.5	–	24.9	–	23.5	24.3
Hypertension (%)	39.1	35.5	51.2	51.7	76.7	78.4	67.2
Diabetes mellitus (%)	8.7	22.2	21.6	13.6	26.7	37.4	37.9
Hyperlipidemia (%)	28.3 (>200 mg/dL)	19.1 (≧240 mg/dL)	–	51.9 (≧220 mg/dL)	42.4 (≧240 mg/dL)	73.7 (≧160 mg/dL)	–
Carotid plaque (%)	68	50 (≧60 years)	–	–	9 (≧50% stenosis)	–	66
ABI < 0.9 (%)	2.9	–	–	9.6 (≧65 years)	–	6.1	–
Cognitive impairment (%)	13.7^*^	–	18.8^†^	11.7 (≧65 years) ^†^	6^†^	–	20.1^†^

* AD8 test;

† *Mini-Mental State Examination*.

*CTPS, carotid total plaque score; CIMT, carotid intima-media thickness; ABI, ankle-brachial index; BMI, Body mass index; AD8, Eight-item Interview to Differentiate Aging and Dementia*.

Carotid plaque has been reported as a predictor of ischemic stroke or cardiovascular disease (35, 36). Non-invasive ultrasonographic assessments of CIMT and plaque are widely used to evaluate the burden of atherosclerosis and are suitable for large-population studies (37, 38). The percentage of plaque increased with aging. Carotid plaque was found in 36.9% asymptomatic subjects in Taiwan with a mean age of 49 years who received a general physical checkup (39). A Taiwanese community-based study of 533 subjects (49% had hypertension) with a mean age of 64.6 years revealed that 41% had carotid plaque; among these, 19% had high plaque scores (40). Carotid plaque was observed in 66% of 348 subjects with a mean age of 71.9 years in a population-based study in Korea (32). Moderate to severe carotid stenosis (≥50% stenosis) was found in 9% of all subjects in a Chinese community study by Yan et al. (30). In the present study, we found that 68% of the subjects had carotid plaque; among these, 16% had a moderate degree of carotid atherosclerosis (CTPS 5.1–10), requiring more effective control of risk factors and possibly antithrombotic treatment. Severe carotid atherosclerosis (CTPS > 10) was found in 7% of the subjects, requiring long-term antithrombotic treatment to prevent cerebrovascular or cardiovascular events. Prompt medical treatment with regular sonographic follow-up examinations of the three subjects with high-grade stenosis was critical due to the high ischemic stroke risk. Endovascular stenting of the carotid artery might be indicated in case of an ineffective response to medical treatment. One study identified advanced age, male sex, and hyperlipidemia as independent risk factors for carotid atherosclerosis (15). In the present study, factors influencing severe carotid atherosclerosis were found to be age ≥70 years, male sex, history of heart disease, history of hyperlipidemia, elevated blood pressure, and elevated cholesterol level, with a model-fitting predictive power of 0.752 using *C*-statistics. Among these, age was the predominant factor. Positive correlations between age and SBP, CIMT, and CTPS were found, as well as a notable negative correlation between age and cholesterol level. Subjects who were vegetarians had lower cholesterol levels and BMI, and the average age of the vegetarian subjects was lower than that of the non-vegetarian subjects. Although duration of diet was not regularly addressed in previous studies, participants in 22 out of 32 studies had been following a vegetarian diet for more than 1 year, according to a meta-analysis study (16). Long-term consumption of a low-calorie low-protein vegetarian diet is associated with a decrease in multiple cardiovascular risk factors. A vegetarian diet has also been reported to improve lipid profile and therefore benefits CIMT, and this appeared to be correlated with the duration of vegetarian diet (14, 15).

Mental disorders, particularly presenting as anxiety and depression, are common in community (20). Depression was considered the main predictor of suicide ideation in psychiatric patients (41). The BSRS-5 was reported to be a satisfactory instrument for detecting psychiatric morbidity and predicting suicide ideation (19) and has been widely used as a screening tool (or “mood thermometer”) for mental health evaluation in Taiwan (20). The optimal cutoff point of BSRS-5 for distinguishing mood disorder (or suicidal ideation) was reported to be 5/6, although varying cutoff points for different population groups were also suggested (41). Using a BSRS-5 with a cutoff point of 5/6, minor psychiatric morbidity was reported at 8.3% for people aged ≥15 years in a nationwide community survey (20), 27% for a hospital health-screening population (mean age: 54 years) (17), and 47% for healthy workers (mean age: 38), among whom 17% had moderate to severe mood disorders (25). In this study, the incidence of mood disorder was low among recycling volunteers with a mean age of 71 years [11%]. The mild mood disorders were mostly anxiety and insomnia. SBP had a positive linear correlation with the BSRS-5 score and was significantly higher in subjects with moderate mood disorder in the present study. It is unclear whether mood disorder itself causes stress-related elevated blood pressure or this is a possible effect of antihypertensive medications on mental health is unclear. Moderate mood disorders were observed in only 1% of the subjects and occurred less often among those with a higher frequency of weekly recycling activities. The enjoyable experience of working at an environmental station with like-minded neighbors and friends might have resulted in an improvement in mood. Moreover, religious beliefs motivated the subjects to continue with recycling work.

Dementia and mild cognitive impairment are generally under-recognized in the community. The prevalence of mild cognitive impairment among people aged ≥65 years in Taiwan was reported at 18.8% (7). The AD8 contains items that test for memory, orientation, judgment, and function (18) and is a convenient tool for quick cognitive function screening during primary care or a population survey. An AD8 score of >2 indicates impaired cognitive function, with further dementia evaluation recommended. Using the AD8, we found that 13% of the subjects had mild cognitive impairment. After excluding subjects younger than 65 years, the percentage of mild cognitive impairment was 13.7% in those aged ≥65 years and over, which is lower than the aforementioned figure of 18.8%. The sensitivity and specificity of AD8 have been reported to be 85% and 86%, respectively, with an area under the receiver operator characteristic curve of 0.834 (18). Thus, the actual prevalence of mild cognitive impairment might reduce from 13.7% to 11.3% after further evaluation with Mini-Mental State Examination, which is similar to the reported prevalence of 11.7% in people≧65 years by Wang et al. in 2016 (29). Mild cognitive impairment was found in 20% of subjects with a mean age of 71.9 years in a Korean population-based study (32). Advanced age, female sex, and a low educational level were reported to be factors significantly associated with dementia or mild cognitive impairment (7). Similarly, our study found that AD8 score increased with aging. Mild cognitive impairment occurred more often in subjects with illiteracy; however, no difference between male and female subjects was found. History of hypertension and hyperlipidemia were also significant predictors for mild cognitive impairment. The AD8 score had a positive linear correlation with CTPS and subjects with mild cognitive impairment had higher CTPSs. Previous studies have demonstrated that systemic vascular health problem, such as carotid stenosis and carotid stiffness are related to cognitive function even in the subclinical phase (30, 42, 43). Vascular dementia is the second commonest type of dementia in elderly people who have cerebral vascular disease from atherosclerosis. Cognitive impairment and mood disorder usually coexist. Patients with dementia may experience depression during the course of the disease. Although patients with mood disorder have a higher risk of mild cognitive impairment (44), certain patients with mood-associated cognitive impairment might see their condition evolve into dementia. The factors related to lower prevalence of mild cognitive impairment in the present study were multifaceted, including a healthier lifestyle with a better mood condition, a higher prevalence of vegetarian diet to reduce the vascular risk of dementia, and regular outdoor work activity at the environmental station to prevent social withdrawal. Moreover, sorting and classifying recyclable items provide an opportunity for elderly people to exercise recognition and judgment and engage in physical activity.

A framework for ideal cardiovascular health (also known as Life's Simple 7) on a population-wide basis for healthy heart and brain function and aging was recommended by the American Heart Association in 2010 (45). Life's Simple 7 comprises three behavioral markers (namely, diet, physical activity, and smoking) and four biological markers (namely, BMI, blood pressure, total cholesterol, and fasting blood glucose). Better cardiovascular health may promote enduring cognitive health (46). The lifestyles of the recycling volunteers in the present study appeared similar to that described in the framework for ideal cardiovascular health. Given the increasing awareness of the waste crisis, recycling is meaningful for people of Taiwan. People are willing to separate recyclable waste in their homes to feel as though they are contributing to saving the planet. Recycling programs implemented by the Taiwanese government have been subject to widespread promotion from the community level to household level. Elderly people who engage in recycling programs as volunteers are appreciated by the government and religious groups. In addition to the contribution of such activity to the environment, benefits to personal health from working at environmental stations as volunteers, particularly alongside a vegetarian diet, were recognized in this study. The results of this study could inspire more people to volunteer in such a capacity.

The present study had some limitations. First, the one-touch finger blood sugar and cholesterol tests are not as accurate as tests performed in a hospital laboratory. The low-density lipoprotein cholesterol, which is more correlated with risk of atherosclerosis, was not measured. Second, medical history and vegetarian diet were self-reported by the subjects, without further confirmation. Third, the BSRS-5 and AD8 were not administered by the same team members during each community visit, and thus interrater deviations occurred even though all members received instructions and training before each test. Fourth, this was a cross-sectional study. Volunteers with abnormal results were recommended to visit specialist physicians for a detailed investigation. A causal relationship between recycling work and mood disorder or cognitive function cannot be determined in a cross-sectional study. However, the conclusion of this study is reasonable based on the study results. Further research in the form of longitudinal follow-up studies could help to clarify the benefits of this vascular, cognitive and psychomental survey.

In conclusion, subclinical carotid atherosclerosis was common in elderly recycling volunteers, with 23% having moderate to severe stenosis. Vegetarians had a reduced risk of atherosclerosis. The low incidence of moderate mood disorder might indicate that recycling work enhances psychomental health. In addition, a healthier lifestyle, better mood condition, and vegetarian diet might contribute to lower incidence of mild cognitive impairment.

## Author Contributions

G-CC drafted the initial manuscript. G-CC, P-YC, Y-CS, C-LH, F-YY, P-JH, and S-KL participated in the health examination and collected data; S-KL designed the study, conducted data analysis, prepared the figure, and revised the manuscript. All authors have contributed to manuscript revision, read, and approved the submitted version.

### Conflict of Interest Statement

The authors declare that the research was conducted in the absence of any commercial or financial relationships that could be construed as a potential conflict of interest.
